# Immunomethylomic approach to explore the blood neutrophil lymphocyte ratio (NLR) in glioma survival

**DOI:** 10.1186/s13148-017-0316-8

**Published:** 2017-02-02

**Authors:** John K. Wiencke, Devin C. Koestler, Lucas A. Salas, Joseph L. Wiemels, Ritu P. Roy, Helen M. Hansen, Terri Rice, Lucie S. McCoy, Paige M. Bracci, Annette M. Molinaro, Karl T. Kelsey, Margaret R. Wrensch, Brock C. Christensen

**Affiliations:** 1 Department of Neurological Surgery, University of California San Francisco, 1450 3rd Street, San Francisco, CA 94158-0520 USA; 2 Department of Biostatistics, University of Kansas Medical Center, Kansas City, KS 66160 USA; 3 Department of Epidemiology and Biostatistics, University of California San Francisco, San Francisco, CA 94158 USA; 4 Helen Diller Family Comprehensive Cancer Center, University of California San Francisco, San Francisco, CA 94158 USA; 5 Computational Biology Core, University of California San Francisco, San Francisco, CA 94158 USA; 6 Department of Epidemiology, Department of Pathology and Laboratory Medicine, Brown University, Providence, RI 02912 USA; 7 Departments of Epidemiology, Pharmacology & Toxicology, and Community and Family Medicine, Geisel School of Medicine at Dartmouth College, Lebanon, NH 03756 USA

**Keywords:** Glioma, DNA methylation, Neutrophil lymphocyte ratio, Systemic inflammation, Immunomethylomics

## Abstract

**Background:**

Differentially methylated regions (DMRs) within DNA isolated from whole blood can be used to estimate the proportions of circulating leukocyte subtypes. We use the term “immunomethylomics” to describe the application of these immune lineage DMRs to studying leukocyte profiles. Here, we applied this approach to peripheral blood DNA from 72 glioma patients with molecularly defined brain tumors, representing common patient groups with defined characteristic survival times and risk factors. We first estimated the proportions of leukocyte subtypes in samples using deconvolution algorithms with reference DMR libraries from isolated leukocyte populations and Illumina 450K DNA methylation data. Then, we calculated the neutrophil to lymphocyte ratio (NLR) using methylation-derived cell composition estimates (mdNLR). The NLR is considered an indicator of immunosuppressive cells in cancer patients.

**Results:**

Elevated mdNLR scores were observed in glioma patients compared to mdNLR values of published controls. Significantly decreased survival times were associated with mdNLR ≥ 4.0 in Cox proportional hazards models adjusted for age, gender, tumor grade, and molecular subtype (HR 2.02, 95% CI, 1.11–3.69). We also identified five myeloid-related CpGs that were highly correlated with the mdNLR (adjusted *R* ^2^ ≥ 0.80). Each of the five myeloid CpG loci was associated with survival when adjusted for the above covariates and offer a simplified approach for utilizing fresh or archived peripheral blood samples for interrogating a very small number of methylation markers to estimate myeloid immune influences in glioma survival.

**Conclusions:**

The mdNLR (based on DNA methylation) is a novel candidate methylation biomarker that represents immunosuppressive myeloid cells within the blood of glioma patients with potential application in clinical trials and future epidemiologic studies of glioma risk and survival.

**Electronic supplementary material:**

The online version of this article (doi:10.1186/s13148-017-0316-8) contains supplementary material, which is available to authorized users.

## Background

About 14,000 Americans are diagnosed each year with glioma, the most common primary malignant brain tumor [1]. Traditional histopathological criteria, including age and certain tumor markers, are currently being used to assess glioma patient prognosis [2]. Glioblastoma (GBM) patients, classified by the World Health Organization (WHO) as grade IV glioma, have a dismal prognosis with an estimated median survival of only 14.6 months. Younger patients and those with isocitrate dehydrogenase (*IDH*) mutated tumors have more favorable survival. The standard therapies for high-grade glioma, which include surgery, temozolomide (TMZ) chemotherapy, and radiation, have led to relatively modest improvements in survival [3]. Previously, we demonstrated that three key molecular features of glioma, telomerase (*TERT*) promoter mutation, *IDH* mutation, and 1p/19q codeletion, are sufficient to create an integrated molecular classification that defines five principal groups of glioma with characteristic distributions of age at diagnosis, clinical behavior, acquired genetic alterations, and associated germline variants [4]. Among these groups, *IDH* mutant only and *TERT* mutant only tumors are the most common and comprise about 75% of adult glioma patients [4].

While the molecular classification of tumors has substantially improved our understanding of glioma prognosis, immune factors are notably absent in existing prognostic models. This omission is significant as immune evasion is a recognized hallmark of cancer [5], and there is abundant evidence that glioma patients suffer systemic immune defects, with the most profound alterations occurring in GBM patients [6–10]. Recent studies have emphasized the important role of developmentally immature and aberrantly activated myeloid-derived cells as contributing to cancer immunosuppression and adversely affecting patient survival [11–13]. Furthermore, immune interventions represent a potentially powerful new therapeutic approach in glioma [14, 15].

The best methods to assess altered myeloid populations or systemic immunosuppression, more generally, are still evolving [16] and, as a result, large-scale studies are lacking. However, the peripheral blood neutrophil to lymphocyte ratio (NLR), which can be derived using the common five-part white blood cell differential (neutrophils, basophils, eosinophils, monocytes, lymphocytes), has emerged as a surprisingly robust marker of cancer associated inflammation [17]. Increases in the blood NLR have been remarkably consistent in their association with poor cancer survival. A recent meta-analysis including 100 independent studies encompassing over 40,000 subjects demonstrated that an elevated NLR was a statistically significant predictor of poor overall survival, cancer-specific survival, as well as progression free and disease free survival, even after adjustment for established risk predictors [18]. There are four studies showing shorter survival times in glioma patients with an elevated NLR [19–22]. Importantly, however, no study has taken into account the molecular features of glioma in conjunction with the NLR or other immune factors.

In this study, our goal was to apply a new epigenetic approach to immune profiling to explore myeloid-related blood markers in glioma survival. Specifically, we examined the peripheral blood DNA methylation status of glioma cases using bioinformatic algorithms that deconvolute the complex methylation signature of whole blood into its component cell compartments [23–26]. This approach to immune studies is based on recent epigenetic discoveries showing that differentially methylated regions (DMRs) provide highly specific and quantitative markers of immune cell profiles [27, 28]. Recently, we developed and validated an algorithm to estimate the NLR from 450K methylation data (methylation-derived NLR; mdNLR) [29]. Our results showed strong agreement between mdNLR and cytological NLR, and elevated mdNLR was significantly associated with diminished patient survival times in head and neck squamous cell carcinoma and bladder cancer, as well as breast and ovarian cancer risk [29], paralleling the now considerable literature on the relationship between cytological NLR and cancer survivorship [18]. Here, we studied the association of the mdNLR with survival among glioma patients.

Because altered myeloid differentiation is implicated in immune alterations in glioma, we also explored the idea that associations of mdNLR in glioma may be linked to myeloid-specific developmental CpG loci. We identified myeloid versus lymphoid specific CpGs on the 450K array that strongly correlate with the mdNLR. This provides important evidence that the NLR is a surrogate marker of myeloid suppression. Consequently, both the mdNLR and the myeloid single CpGs are potential markers of skewed myeloid profiles that may be useful in characterizing immune defects associated with survival in glioma.

## Methods

### Patient samples

Patients were chosen from the University of California San Francisco (UCSF) Adult Glioma Study (AGS) who had both archival blood and tumor marker data [30]. AGS participants represent primary glioma patients; no recurrent or secondary GBM cases are included. Seventy-two cases were selected from the two most prevalent molecular subtypes of glioma [4] (i.e., cases with *IDH* mutation only or *TERT* promoter mutation only). Samples from cases aged 40 to 59 were selected as follows: all available non-GBMs and IDH-only GBMs were included. TERT-only GBMs were chosen to match the ages of both the IDH-only GBMs and the TERT-only non-GBMs. Blood samples were collected from patients a median of 100 days after they were histologically diagnosed. Clinical information was collected on patient treatments including temozolomide (TMZ) chemotherapy, radiation therapy, extent of surgery, and steroid use at the time of blood sampling. The anticoagulated whole blood was processed, and DNA was isolated and bisulfite converted as previously described (27).

### Quality control and preprocessing of the DNA methylation data

Illumina 450K arrays were run by the UCSF Human Genomics core. Preprocessing and quality control was accomplished using the *minfi* Bioconductor package [31]. To ensure high-quality methylation data, CpG loci having a sizable fraction (>25%) of detection *p* values above a predetermined threshold (detection *P* > 10E–5) were excluded [32]. Subset Quantile Within Array (SWAN) normalization was performed for type 1/2 probe adjustment [33]. The presence of technical sources of variability induced by plate and/or BeadChip was examined using principal components analysis (PCA), and the top *K* principal components [34] were examined in terms of their association with plate and BeadChip. If plate and/or BeadChip was found to be significantly associated with any of the top *K* principal components, we applied ComBat method [35] for normalization using the *sva* Bioconductor package.

### Cell mixture deconvolution analysis

Using the preprocessed and normalized methylation data, we applied an optimized reference-based cell mixture deconvolution methodology [28] to gain insight into the cellular composition of the samples considered here. Specifically, the proportions of CD4+ T cells, CD8+ T cells, B cells, natural killer (NK) cells, monocytes, and granulocytes were estimated for each sample using the function “estimateCellCounts” in the *minfi* Bioconductor package using an optimized reference library set of CpGs.

### Computing the methylation-derived neutrophil lymphocyte ratio (mdNLR)

Estimation of the mdNLR was carried out as previously described [29]. Briefly, the method requires three main steps: (*i*) identify differentially methylated CpGs among leukocyte subtypes, (L-DMRs), (*ii*) perform cell mixture deconvolution to estimate the proportion of leukocyte subtypes using L-DMRs identified in step 1, and (*iii*) compute the ratio of the predicted proportion of neutrophil granulocytes to lymphocytes. The mdNLR was computed by taking the ratio of predicted granulocyte and lymphocyte fractions, mdNLR_*i*_ $=\frac{\widehat{\omega}\left(\mathsf{Gran}, i\right)}{\widehat{\omega}{\left(\mathsf{Lymph}, i\right)}^{,}}$ *0 ≤* mdNLR_*i*_ *< ∞.* The mdNLR scores are based on beta values using 300 L-DMR CpGs [28]. A publicly available implementation of this method is available in the *IDOL* R package (https://www.r-project.org/).^1^

### Statistical analyses of the mdNLR and clinical outcomes

Associations between mdNLR and clinical covariates were assessed using either logistic regression or linear regression models. Cox proportional hazards regression models were used to examine the association between mdNLR and survival time and were fit using the “coxph” function in the *survival* R package. Survival models were adjusted for established risk predictors and potential confounders, including age, gender, histological subtype (GBM versus non-GBM), and IDH/TERT mutation status (IDH-only mutation versus TERT-only mutation). The proportionality assumption was assessed by plotting the scaled Schoenfeld residuals against time, and the “cox.zph” function in the *survival* R package was used for testing the proportionality of each predictor included in our survival models [36]. In our survival analyses, mdNLR was modeled both as a continuous predictor and by dichotomizing subjects into high and low mdNLR groups. The binary cut point of mdNLR >4 is based on previous studies [19]. We also compared the performance of different survival models that included known risk factors compared with analyses including mdNLR and single locus CpGs. Three metrics were computed using the packages *survival* and *survAUC* to compare the performance of these models: concordance index (c-index), the Gerds and Schumacher Brier score, and the Song and Zhou [37] time-dependent area under the receiver operator characteristic curve (tAUROC) [38]. Log-rank tests were used to judge differences between the experimental and baseline model. The baseline model contained patient age, gender, tumor grade, and mutation status (*TERT* mutant only vs *IDH* mutant only).

### Identification of myeloid-specific single locus markers of the mdNLR

While the mdNLR requires 300 CpGs to estimate the neutrophil lymphocyte ratio (29), we hypothesized that the NLR (and the mdNLR) is a biomarker of the known influx of myeloid-derived suppressor cells into the peripheral blood that occurs with the development of a new cancer (11), and as a result of this, reasoned that there may exist individual influential CpGs arising during myeloid differentiation that could serve as surrogates for the mdNLR. To test this hypothesis, we first sought to identify myeloid-specific markers. The *M* values of 54 samples from the Reinius dataset (excluding the six whole blood samples, GSE35069 [39]) were modeled according to if they were predominantly myeloid or lymphoid cells, adjusting for the proportion of the blood cells in the samples as measured by flow cytometry. The top 100 loci were selected using the RnBeads automatic rank cutoff approach. A second model then evaluated the relationship between the mdNLR as the outcome and the top 100 myeloid-specific loci to obtain a reduced list of methylation-derived mdNLR surrogates. For variance stabilization, beta values were converted to *M* values and were modeled assuming linear, quadratic, and cubic relationships with survival time; we then computed adjusted *R* ^2^ values to assess the correlation of each methylation site. We first modeled the 100 myeloid-specific loci using the methylation data from subjects in this study and then repeated the models in the Hannum [40] [GSE40279] and Liu [41] [GSE42861] blood methylation datasets. For the top 10 models, the adjusted *R* ^2^ ranged between 40–86%. Five loci were consistently found to obtain an adjusted *R* ^2^ over 80% in all three datasets. Each of the five loci was markedly demethylated in myeloid compared to lymphoid cells and stem cells (using ENCODE resources).

## Results

### Neutrophil lymphocyte ratio in glioma patients assessed by immunomethylomics

**Fig. 1:**
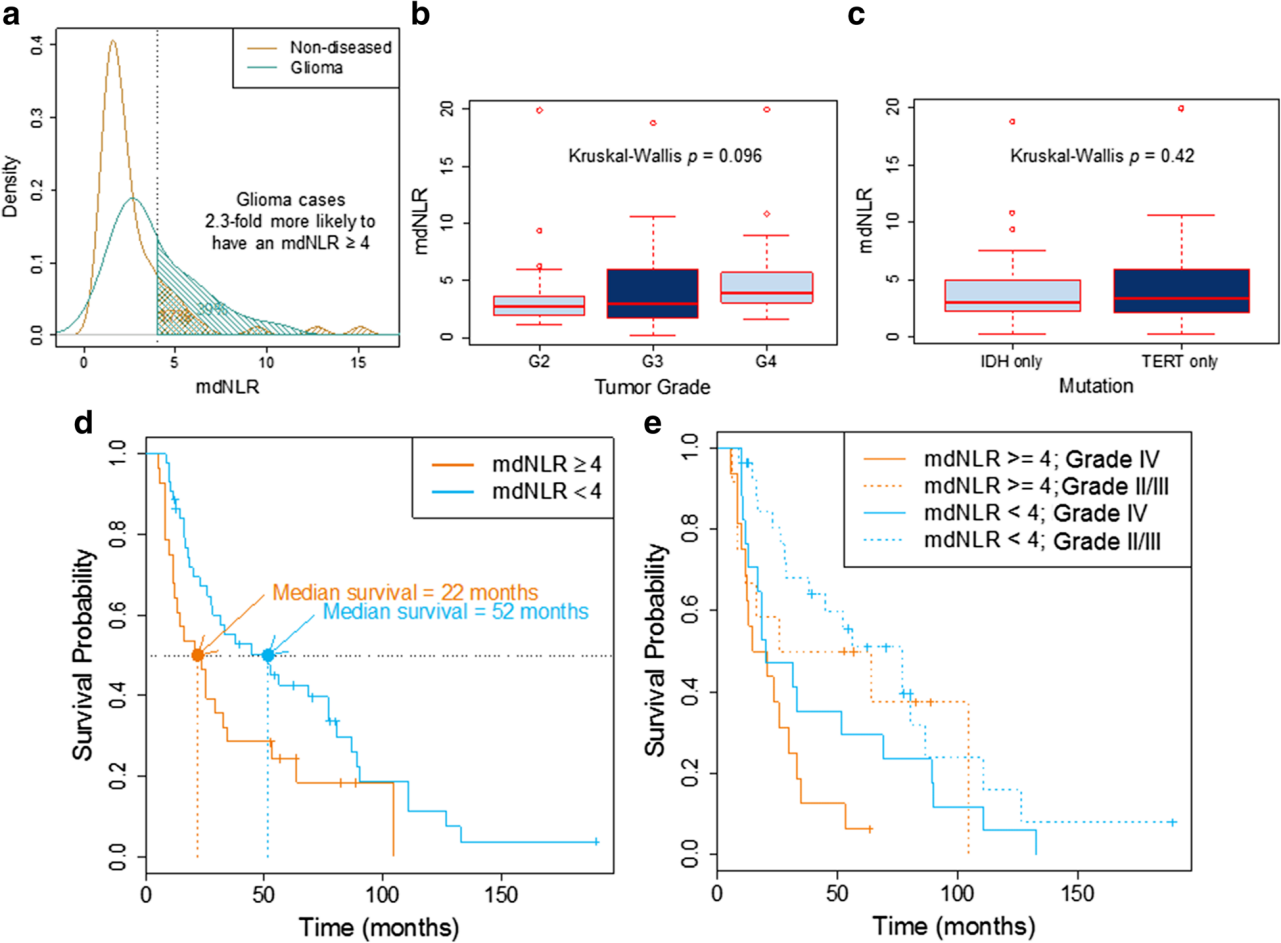
**a** Comparison of the distributions of mdNLR among glioma cases and a non-cancer comparison group. **b** *Boxplot* comparing mdNLR of glioma patients by tumor grade. **c** *Boxplot* comparing mdNLR of glioma patients by tumor molecular subtype. **d** Kaplan-Meier survival curves stratified by mdNLR (<4 vs > = 4) **e** Kaplan-Meier survival curves stratified by histopathology (GBM vs non-GBM) and mdNLR (<4 vs > = 4)

The study sample sizes, clinical characteristics, and available demographic/epidemiological data are given in Table 1. Leukocyte cell composition of whole blood was calculated with our validated algorithm and optimized reference libraries using the IDOL procedure [28], Additional file 1: Figure S1. Combining the myeloid and lymphocytic subtypes allowed the calculation of the mdNLR. The mdNLR scores among glioma cases were then compared with a large public database of blood methylation data collected on 656 non-cancer adults [40]. Figure 1a compares the distributions of mdNLR among glioma cases and the non-cancer comparison group; the median mdNLR of glioma patients was elevated compared to the non-cancer group. Higher glioma tumor grade was associated with increased mdNLR values (panel B), but mdNLR scores were similar among cases whose tumors contained *IDH*1 vs *TERT* promoter mutation (panel C).

**Table 1:** Summary of patient characteristics

Number	72
Median age (IQR)	47 (44, 54) years
Sex	
Male	72%
Female	28%
Histology and grade	
Astro/oligo/oligoastro gr II–III	54%
Glioblastoma multiforme gr IV	46%
Mutation status	
*TERT* promoter only	58%
*IDH*-only	42%
Methylation-derived NLR (mdNLR)	
mdNLR < 4 (%)	61%
mdNLR ≥ 4 (%)	39%
Length of follow-up	5–190 months
Median survival time (IQR)	29 (13, 65) months

### Association of mdNLR with glioma survival times

Median survival in cases with mdNLR < 4 was 52 months compared to those with elevated mdNLR scores; 22 months (Fig. 1d). Kaplan-Meier survival curves were further stratified by histopathology (GBM vs non-GBM) and suggested shorter survival times among GBM cases, although sample sizes are limited (Fig. 1e). Cox proportional hazards models that included known prognostic factors (age, grade, mutation status) indicated significant association of a high mdNLR (>4) with an increased risk of death; HR 2.02, 95% CI, 1.11–3.69, *P* = 0.02 (Table 2). A Cox model including chemotherapy and steroid use suggests that mdNLR is associated with survival time, independent of therapy; HR 1.84, 95% CI, 1.00–3.38, *P* = 0.049 (Additional file 2: Table S1).

**Table 2:** Cox proportional hazards survival models including mdNLR, age, grade, and tumor mutation status

		*n* deceased	Survival years		Univariate models		Multivariate model	
	Number		Mean	Median	HR (95% CI)	*p* value	HR (95% CI)	*p* value
mdNLR < 4 mdNLR > = 4	44 28	35 23	4.8 3.1	4.3 1.8	Referent group 1.78 (1.03–3.07)	0.038	Referent group 2.02 (1.11–3.69)	0.022
GBM non-GBM	33 39	32 26	2.9 5.3	1.7 5.3	Referent group 0.48 (0.28–0.81)	0.006	Referent group 1.06 (0.56–2.00)	0.859
*IDH* only *TERT* only	30 42	18 40	6.6 2.4	7.2 1.4	4.19 (2.34–7.48)	<0.0001	referent group 4.83 (2.35–9.93)	0.00002
Age (continuous)							0.97 (0.92–1.02)	0.274

All covariates modeled met proportionality assumptions

*HR* hazard ratio, *CI* confidence interval

*MdNLR* methylation-derived NLR (neutrophil to lymphocyte ratio)

Glioma grading was based on WHO 2007 criteria; however, since we know *IDH* mutation and 1p19q codeletion status, we can reclassify these cases using the new WHO 2016 brain tumor classification [42]. Based on the WHO 2016 criteria, two anaplastic oligodendroglioma or oligoastrocytomas cases would have been classified as GBM instead of non-GBM due to having evidence of microvascular proliferation. This reclassification would not have substantially altered the results of this analysis.

### Association of single CpG myeloid differentiation loci with mdNLR and survival

**Fig. 2:**
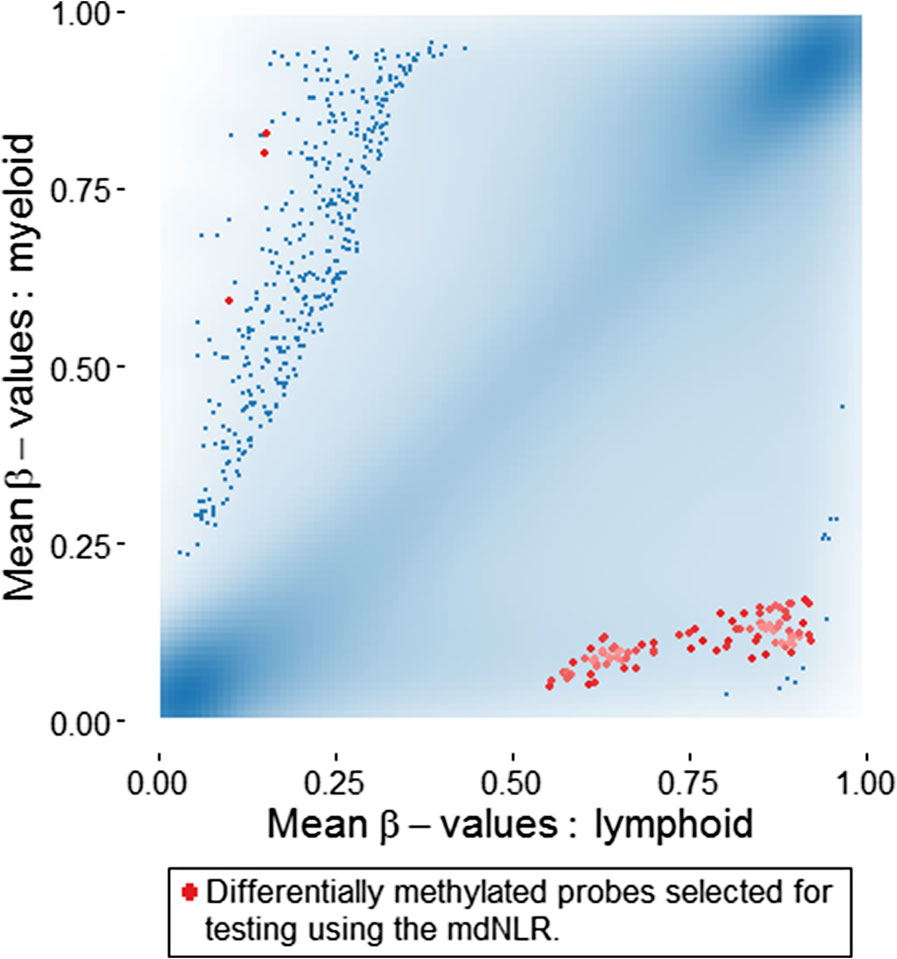
Identification of myeloid and lymphoid specific CpG probes. *Scatterplot* depicting Illumina 450K methylation beta values among isolated lymphocyte subtypes (X-axis: T cells, B cell, NK cells) and myeloid subtypes (Y-axis; granulocytes, monocytes). The *lower right quadrant* identifies loci that are unmethylated in myeloid cells and densely methylated in lymphocytes

**Fig. 3:**
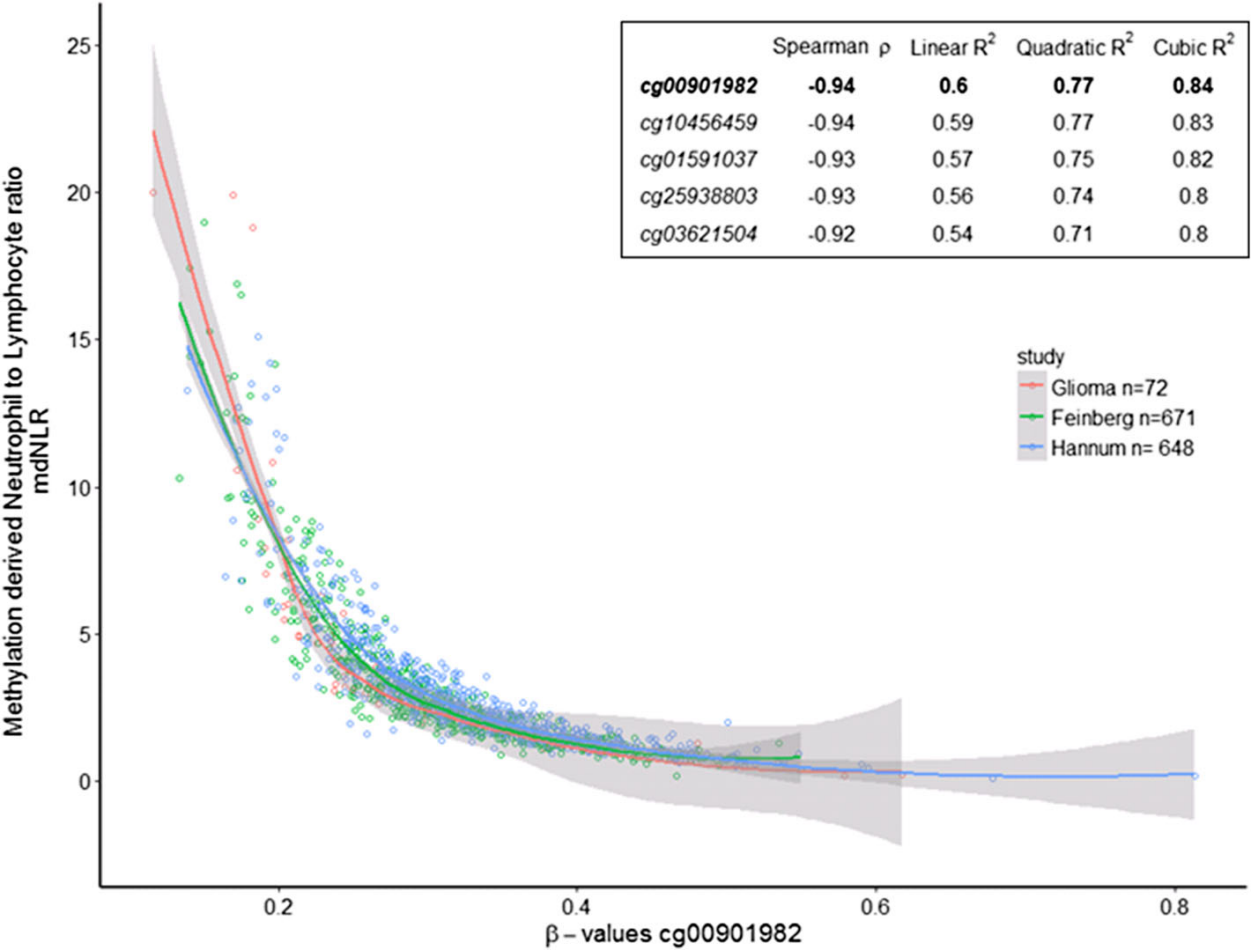
Correlation of myeloid locus with mdNLR

Candidate loci representing myeloid-specific CpGs were identified, and the top 100 included loci hypomethylated in myeloid cells compared to lymphoid cells and only a few loci that were hypermethylated in myeloid cells Fig. 2. Genes associated with these myeloid-specific loci are summarized in Table 3. Five loci were chosen that showed very strong correlation with the mdNLR across three independent blood DNA methylation datasets, Fig. 3. Among the different models examined, the quadratic form best fit the regression of CpG methylation and mdNLR. Table 4 describes the methylation levels of these five loci according to glioma patient characteristics (tumor grade, mutation status, NLR status). The data indicate the strong association of each individual loci with patient NLR status.

**Table 3:** Top five myeloid-specific loci

	Chromosome	MAPINFO hg19 location	Strand	Annotated gene	Gene located on the same or opposite transcription strand	SNP 10 bases to hybridization	MAF	Genomic context	Infinium	Enhancer
cg25938803	chr2	43767347	+	THADA	Opposite	rs183844032^a^	0.0002	Body	II	
Sequence^b^										
GCACTACAGCCAGTCACCAGCAATGACTGCAAGTAACTCTAGGACACTGACGCCTATTTGATTTGGAAGAGAATAAGGAACATAATGATGCCTGAAATGTC										
cg00901982	chr2	70257298	−	PCBP1-AS1	Same	rs533928090	0.0002	Body	II	
Sequence^b^										
GACATTTCAGGCATCATTATGTTCCTTATTCTCTTCCAAATCAAATAGGCGTCAGTGTCCTAGAGTTACTTGCAGTCATTGCTGGTGACTGGCTGTAGTGC										
cg01591037	chr12	15134481	−	PDE6H	Opposite	rs144778897^a^	0.001597	3UTR	II	
Sequence^b^										
GACATTTCAGGCATCATTATGTTCCTTATTCTCTTCCAAATCAAATAGGCGTCAGTGTCCTAGAGTTACTTGCAGTCATTGCTGGTGACTGGCTGTAGTGC										
cg10456459	chr12	22843015	+	ETNK1	Same	rs373083641	0.0002	3UTR	II	True
Sequence^b^										
GCACTACAGCCAGTCACCAGCAATGACTGCAAGTAACTCTAGGACACTGACGCCTATTTGATTTGGAAGAGAATAAGGAACATAATGATGCCTGAAATGTC										
cg03621504	chr12	116571240	+	MED13L	Opposite	N/A	N/A	Body	II	
Sequence^b^										
GCACTACAGCCAGTCACCAGCAATGACTGCAAGTAACTCTAGGACACTGACGCCTATTTGATTTGGAAGAGAATAAGGAACATAATGATGCCTGAAATGTC										

*MAF* minor allele frequency

^a^SNP on hybridization site

^b^Sequence corresponding to 50 bases upstream and 50 bases downstream of the CpG location based on the GRCh37/hg19 build

**Table 4:** NLR associated single CpG loci median (IQR) beta values in glioma patient subgroups

CpG loci	NLR status			Tumor grade			Mutation status		
	NLR high	NLR low	*P*	II/III	IV	*P*	*IDH1*	*TERT*	*P*
cg25938803	24.18 (22.72, 26.16)	33.55 (31.25, 39.00)	<0.001	28.33 (24.67, 32.50)	31.40 (26.20, 38.09)	0.1	30.73 (26.13, 34.66)	30.31 (24.44, 35.24)	0.8
cg00901982	20.63 (19.11, 22.61)	30.29 (26.67, 34.76)	<0.001	24.19 (21.17, 26.97)	27.71 (23.16, 34.32)	0.04	26.71 (23.00, 30.73)	24.69 (21.12, 32.06)	0.5
cg01591037	25.48 (23.58, 27.93)	33.87 (32.13, 39.76)	<0.001	30.14 (25.84, 33.75)	32.66 (27.94, 38.44)	0.1	32.01 (27.50, 36.35)	31.03 (25.84, 36.50)	0.6
cg10456459	26.49 (24.34, 28.20)	38.02 (35.68, 43.86)	<0.001	32.19 (27.47, 35.90)	36.44 (27.72, 44.06)	0.05	36.14 (28.20, 39.10)	32.97 (27.36, 39.06)	0.5
cg03621504	20.23 (18.28, 21.37)	26.25 (23.76, 30.42)	<0.001	22.47 (20.74, 26.12)	23.76 (21.39, 29.92)	0.2	23.66 (20.89, 26.40)	22.90 (20.88, 26.56)	0.8

Note: Beta values are represented as percentages (beta values times 100). Median differences between groups were tested using a Mann-Whitney *U* test

We compared the performance of survival models that contain the mdNLR and found a significant difference from the base model which did not contain the mdNLR and a modest increase in the concordance score and Brier score (Table 5). Models that individually included one of each of the five myeloid-specific differentiation CpGs revealed that the loci were significant compared to the base model and produced concordance and Brier’s scores equivalent to the mdNLR. As similar results were found when any of the five loci were included, we included only one of them (cg00901982) in Table 5. We also examined models containing the mdNLR in addition to each of the five loci (Table 6). As expected, when both variables are included in the models, little additional variance is explained.

**Table 5:** Cox proportional hazards survival models including, age, sex, grade, mutation status, and either mdNLR or cg00901982 (linear and quadratic terms)

			Baseline model	Baseline + mdNLR	Baseline + CpG	Baseline + CpG + CpG^2^
	*n* (%)	Median (IQR)	HR (95%CI)	HR (95%CI)	HR (95%CI)	HR (95%CI)
Age		47 (44, 54)	0.99 (0.94, 1.05)	0.97 (0.92, 1.03)	0.99 (0.94, 1.04)	0.97 (0.92, 1.03)
Female	20 (28)		Referent group	Referent group	Referent group	Referent group
Male	52 (72)		0.75 (0.41, 1.38)	0.74 (0.40, 1.35)	0.74 (0.40, 1.35)	0.69 (0.37, 1.26)
mdNLR >4	28 (39)			Referent group		
mdNLR ≤4	44 (61)			0.49 (0.27, 0.90)		
IDH only	30 (42)		Referent group	Referent group	Referent group	Referent group
TERT only	42 (58)		3.96 (1.98, 7.94)	4.56 (2.20, 9.43)	4.25 (2.09, 8.64)	4.65 (2.25, 9.62)
GBM	33 (46)		Referent group	Referent group	Referent group	Referent group
Non-GBM	39 (54)		0.92 (0.50, 1.71)	1.02 (0.54, 1.92)	0.98 (0.52, 1.82)	0.90 (0.48, 1.70)
cg00901982*		26.1 (21.4, 31.2)			0.80 (0.52, 1.22)	0.36 (0.04, 3.15)
cg00901982^2^*						29 (3.52, 237)
Concordance			0.71 (SE = 0.04)	0.73 (SE = 0.04)	0.72 (SE = 0.04)	0.74 (SE = 0.04)
Brier score			0.1508	0.1506	0.1511	0.1468
Lrtest vs baseline model				**0.02**	0.29	**0.01**
Lrtest vs baseline + mdNLR model					**<0.0001**	0.06
Lrtest model linear (CpG) vs quadratic (CpG + CpG^2^) model						**0.01**

*p* values <0.05 are highlighted in bold font

All covariates modeled met proportionality assumptions

*HR* hazard ratio, *CI* confidence interval, *mdNLR* methylation-derived neutrophil lymphocyte ratio, *Lrtest* likelihood ratio test

*Per every 10% increase in methylation

**Table 6:** Cox proportional hazards survival models including, age, sex, grade, mutation status, mdNLR and cg00901982 (linear and quadratic terms)

			Baseline model	Baseline + NLR	Baseline + mdNLR + CpG	Baseline + mdNLR + CpG + CpG^2^
	*n* (%)	Mean (sd)	HR (95%CI)	HR (95%CI)	HR (95%CI)	HR (95%CI)
Age		47 (44, 54)	0.99 (0.94, 1.05)	0.97 (0.92, 1.03)	0.97 (0.92, 1.03)	0.97 (0.92, 1.02)
Female	20 (27.8)		Referent group	Referent group	Referent group	Referent group
Male	52 (72.2)		0.75 (0.41, 1.38)	0.74 (0.4, 1.35)	0.74 (0.41, 1.36)	0.7 (0.38, 1.29)
mdNLR ≥4	28 (38.9)			Referent group	Referent group	Referent group
mdNLR <4	44 (61.1)			0.49 (0.27, 0.9)	0.40 (0.17, 0.92)	0.69 (0.26, 1.81)
IDH only	30 (41.7)		Referent group	Referent group	Referent group	Referent group
TERT only	42 (58.3)		3.96 (1.98, 7.94)	4.56 (2.20, 9.43)	4.49 (2.16, 9.35)	4.73 (2.26, 9.88)
GBM	33 (45.8)		Referent group	Referent group	Referent group	Referent group
Non-GBM	39 (54.2)		0.92 (0.50, 1.71)	1.02 (0.54, 1.92)	1.00 (0.53, 1.90)	0.92 (0.48, 1.76)
cg00901982*		26.1 (21.4, 31.2)			1.20 (0.72, 1.99)	0.91 (0.03, 24.7)
cg00901982^2^*						15.9 (1.12, 225)
Concordance			0.71(SE = 0.04)	0.73(SE = 0.04)	0.74(SE = 0.04)	0.74(SE = 0.04)
Brier score			0.1508	0.1506	0.1504	0.1473
Lrtest vs baseline model				**0.02**	0.06	**0.02**
Lrtest vs baseline + mdNLR model					0.49	0.13
Lrtest model linear (CpG) vs quadratic (CpG + CpG^2^) model						0.06

*p* values <0.05 are highlighted in bold font

All covariates modeled met proportionality assumptions

*HR* hazard ratio, *CI* confidence interval, *mdNLR* methylation-derived neutrophil lymphocyte ratio, *Lrtest* likelihood ratio test

*Per every 10% increase in methylation

## Discussion

Shifts in the distribution and numbers of blood leukocytes as well as the emergence of aberrant myeloid cells with immunosuppressive properties are important predictors of cancer patient survival [11–13]. The simple NLR in the whole blood has received a great deal of attention as a replicated marker of cancer inflammation linked to poor survival [18]. Because the NLR reflects the relative balance of the myeloid and lymphocytic lineages in peripheral blood, it is sensitive to the altered myelopoiesis arising in chronic inflammation and cancer. A main finding of this study is that DMRs that distinguish leukocyte subtypes can be used to estimate the NLR ratio and that this epigenetically derived metric, like the cytological NLR, is associated with glioma occurrence and survival times. Our observation of elevated mdNLR values in GBM patients compared to controls is consistent with previous studies [18]. Although the mdNLR is less dramatically elevated in non-GBM compared with GBM cases, the data suggests alterations in some lower grade patients. Our sample of glioma patients was restricted to tumor subtypes containing either *IDH* or *TERT* mutations exclusively. After adjustments for these molecular features and other prognostic factors, the elevated mdNLR was a significant prognostic indicator of shorter survival times. Thus, the immunomethylomic approach to the evaluation of the NLR holds considerable promise in immune profiling. Currently, there is intense interest in multiscale assessment of immune function in cancer patients receiving traditional treatments and new immunotherapies [43]. Immunomethylomics can readily provide cell ratios as in the mdNLR and has the potential to identify aberrant epigenetic subsets of immune cells.

When we evaluated the performance of multivariate survival models with or without the mdNLR, we found a significant but modest improvement of model fit by inclusion of the mdNLR. This is not unexpected as the molecular subtypes selected for the current study represent very divergent prognostic groups. Survival for patients with *IDH*-only mutant glioma is much longer compared with those harboring *TERT* promoter mutation only tumors. Thus, survival models containing these mutation factors explain a large degree of variation in survival times and improvements in predictive performance above the base model were modest in size. This is not uncommon in cancer studies. Nonetheless, the direction of the association of the mdNLR with survival is consistent with previous studies in glioma and other solid tumors that implicate myeloid factors in cancer inflammation. However, a caveat to this interpretation is that decreases in lymphocytes could drive increases in the mdNLR without alterations in the myeloid compartment. While the mdNLR is affected by either increased myeloid or decreased lymphocyte counts, the individual myeloid-specific differentiation loci are less susceptible to this effect of lymphocyte depletion. It is of interest therefore, that each of the five myeloid-specific loci performed similarly to the mdNLR and produced largely comparable performance metrics in multivariate analyses. 

The single CpG sites that correlated with the mdNLR are not located in classic promoter regions and their functional significance is unknown. Further work is needed to develop a deeper understanding of the potential biological significance of the five loci. It will be of interest to know whether the single CpG myeloid loci identified in this study are related to the immature and aberrant phenotype of MDSCs. The current state of immunomethylomics does not allow for the direct estimation of MDSCs that are hypothesized to be the drivers of immunosuppression associated with elevated NLR. Thus, future discovery studies of the methylomes of isolated MDSCs that identify their unique DMR repertoire are needed. Specific MDSC defining DMRs can then be added to the reference libraries of normal neutrophil and monocyte cell types and used for cell profiling. Until that time, the current markers of leukocytes, mdNLR, and myeloid differentiation are easily implemented in clinical studies and large population studies. Unprocessed peripheral blood and archival samples are suitable for immunomethylomic profiling. The single CpG myeloid differentiation markers can be used in single locus quantitative assay formats without the requirement for extensive array-based analysis.

## Conclusions

The ratio of neutrophils to lymphocytes in blood has been associated with immune suppression and decreased survival times in multiple solid tumors. Based on immune cell-specific DMRs and validated cell deconvolution algorithms, we estimated the NLR in blood from glioma patients and found elevated mdNLR values in glioma patients compared to a published control group. The patient mdNLR scores were increased in patients with grade IV tumors compared with grade II/III. High mdNLR scores were significantly associated with shorter survival times. We explored the contribution of myeloid differentiation loci in driving the mdNLR and identified candidate single (myeloid-associated) gene loci that were highly correlated with the mdNLR. These loci likely represent myeloid differentiation-specific demethylation events, consistent with the hypothesis that the NLR and mdNLR are biomarkers of myeloid suppression. Individual myeloid differentiation loci were tested in multivariate survival analyses and found to perform as well as the mdNLR in improving survival predictions. Single myeloid differentiation loci provide a simpler and cheaper alternative to the mdNLR, which requires complex array data. Immunomethylomics may be an alternative to conventional cell analysis in profiling glioma risk and survival factors.

## Additional Files

Additional file 1: Figure S1. Leukocyte cell composition of the whole blood calculated with our validated algorithm and optimized reference libraries using the IDOL procedure. (See Additional file 1: Figure S1.png). (PNG 17 kb)
Additional file 2: Table S1. Cox proportional hazards survival models including mdNLR, age, grade, mutation status, and chemotherapy and dexamethasone. (see Additional file 2: Table S1.xlsx). (XLS 7 kb)

## Abbreviations

AGS: Adult Glioma Study
DMR: Differentially methylated region
GBM: Glioblastoma
HR: Hazard ratio
*IDH*: Isocitrate dehydrogenase
mdNLR: Methylation-derived neutrophil lymphocyte ratio
NLR: Neutrophil lymphocyte Ratio
*TERT*: Telomerase reverse transcriptase
TMZ: Temozolomide

## Footnotes

1 The IDOL R-package has been submitted to the Comprehensive R Archive Network (CRAN) and is available through Github. Software is also available by request.
