# Supplementary figures and images for: Immunomethylomic approach to explore the blood neutrophil lymphocyte ratio (NLR) in glioma survival
Source: Clin Epigenetics. 2017 Feb 2;9:10. doi: 10.1186/s13148-017-0316-8 (PMC5288996; doi:10.1186/s13148-017-0316-8)

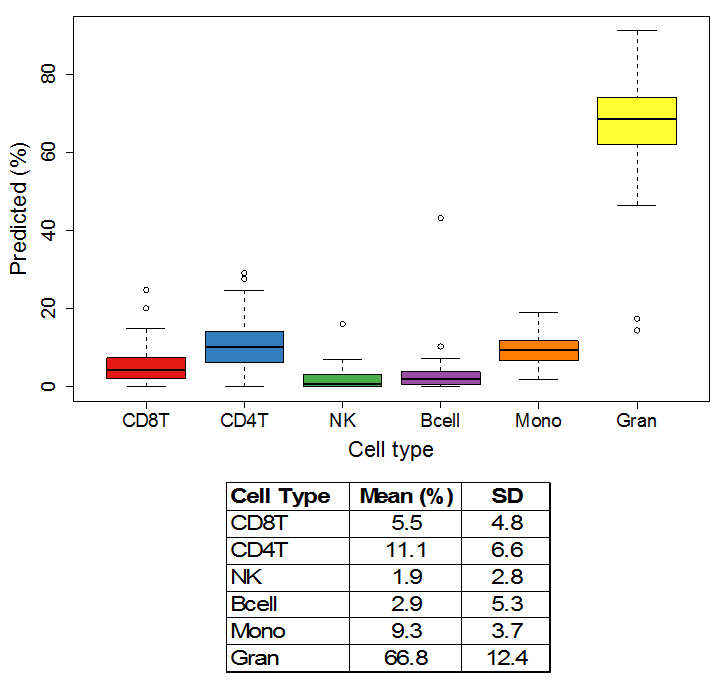

Supplement: Additional file 1: Figure S1. — Leukocyte cell composition of the whole blood calculated with our validated algorithm and optimized reference libraries using the IDOL procedure. (See Additional file 1: Figure S1.png). (PNG 17 kb) [file 13148_2017_316_MOESM1_ESM.png]
